# 72-h diurnal RNA-seq analysis of fully expanded third leaves from maize, sorghum, and foxtail millet at 3-h resolution

**DOI:** 10.1186/s13104-020-05431-5

**Published:** 2021-01-14

**Authors:** Xianjun Lai, Claire Bendix, Yang Zhang, James C. Schnable, Frank G. Harmon

**Affiliations:** 1 Center for Plant Science Innovation & Department of Agronomy and Horticulture, University of Nebraska-Lincoln, Lincoln, 68588 USA; 2 College of Agricultural Sciences, Xichang University, Liangshan, 615000 China; 3 Department of Plant & Microbial Biology, University of California, Berkeley, CA 94720 USA; 4 Plant Gene Expression Center, USDA-ARS, Albany, 94710 USA

**Keywords:** Diurnal rhythms, Fastq file, Foxtail millet, Maize, Panacoid grasses, Poaceae; RNA-seq, Sorghum

## Abstract

**Objectives:**

The purpose of this data set is to capture the complete diurnal (i.e., daily) transcriptome of fully expanded third leaves from the C4 panacoid grasses sorghum (*Sorghum bicolor*), maize (*Zea mays*), and foxtail millet (*Setaria italica*) with RNA-seq transcriptome profiling. These data are the cornerstone of a larger project that examined the conservation and divergence of gene expression networks within these crop plants. This data set focuses on temporal changes in gene expression to identify the network architecture responsible for daily regulation of plant growth and metabolic activities. The power of this data set is fine temporal resolution combined with continuous sampling over multiple days.

**Data description:**

The data set is 72 individual RNA-seq samples representing 24 time course samples each for sorghum, maize, and foxtail millet plants cultivated in a growth chamber under equal intervals of light and darkness. The 24 samples are separated by 3-h intervals so that the data set is a fine scale 72-h analysis of gene expression in the leaves of each plant type. FASTQ files from Illumina sequencing are available at the National Center for Biotechnology Information Sequence Read Archive.

## Objective

The purpose of this data set is to identify transcripts with diurnal (i.e. daily) patterns of regulation within the C4 panacoid grasses sorghum (*Sorghum bicolor*), maize (*Zea mays*), and foxtail millet (*Setaria italica*) with RNA-seq transcriptome profiling. These data are the cornerstone of a larger project that examined the conservation and divergence between sorghum, maize and foxtail millet of circadian clock and diurnal gene expression networks and their local DNA regulatory elements [1]. The working hypothesis motivating the project is critical regulatory features are retained through evolution. Therefore, these features can be discovered by finding transcriptome attributes shared between related, but evolutionarily distant, plant species. The power of this data set is combined fine temporal resolution of sampling and continuous sampling over a 72-h period. Sorghum, maize, and foxtail millet are studied because each is from the panacoid family of grasses, which perform C4 photosynthesis, and is a major crop plant worldwide. This data set focuses on temporal changes in gene expression to identify the network architecture for daily regulation of plant growth and metabolic activities.

## Data description

The data set is 72 individual RNA-seq samples (Table 1) representing 24 fully expanded third leaf samples each for sorghum (BTx623 inbred), maize (B73 inbred), and foxtail millet (Yugu1 accession) plants cultivated in a growth chamber under 12 h light (440 μmol m^−1^ s^−1^ of photosynthetically active radiation provided by cool white fluorescent bulbs) and 12 h darkness for 15 days (sorghum, maize) or 20 days (foxtail millet). Light periods were 26–28 °C and dark periods were 22 °C. Samples were taken at 3-h intervals for a total of 72-h. Sampling began at 3 h from when lights were turned on at the beginning of the first collection day. Quality of total RNA extracted from samples was confirmed by Bioanalyzer (Agilent). Strand-specific RNA-seq libraries were constructed from mRNA, isolated from total RNA by poly(A) selection, as described previously [2]. Multiplexed libraries were paired-end sequenced with 50 cycles (PE50) by Illumina HiSeq 2500 sequencing at the Illumina Sequencing Genomics Resources Core Facility at Weill Cornell Medical College, Ithaca, NY. The average total reads/mapped reads for sorghum, maize and foxtail millet samples was 21,909,425/18,716,318, 20,791,418/15,401,114 and 21,482,551/20,177,538, respectively. The 72 demultiplexed FASTQ files from Illumina sequencing were deposited at the National Center for Biotechnology Information (NCBI) Sequence Read Archive under accession number PRJNA616061.

**Table 1:** Overview of data files/data sets

Label	Name of data file/data set	File types (file extension)	Data repository and identifier (DOI or accession number)
Data file 1	RNA-seq of B73: fully expanded third leaves 3 h	.fastq	NCBI Sequence Read Archive (http://identifiers.org/ncbi/insdc.sra:SRR11442908)
Data file 2	RNA-seq of B73: fully expanded third leaves 6 h	.fastq	NCBI Sequence Read Archive (http://identifiers.org/ncbi/insdc.sra:SRR11442907)
Data file 3	RNA-seq of B73: fully expanded third leaves 9 h	.fastq	NCBI Sequence Read Archive (http://identifiers.org/ncbi/insdc.sra:SRR11442896)
Data file 4	RNA-seq of B73: fully expanded third leaves 12 h	.fastq	NCBI Sequence Read Archive (http://identifiers.org/ncbi/insdc.sra:SRR11442885)
Data file 5	RNA-seq of B73: fully expanded third leaves 15 h	.fastq	NCBI Sequence Read Archive (http://identifiers.org/ncbi/insdc.sra:SRR11442874)
Data file 6	RNA-seq of B73: fully expanded third leaves 18 h	.fastq	NCBI Sequence Read Archive (http://identifiers.org/ncbi/insdc.sra:SRR11442859)
Data file 7	RNA-seq of B73: fully expanded third leaves 21 h	.fastq	NCBI Sequence Read Archive (http://identifiers.org/ncbi/insdc.sra:SRR11442848)
Data file 8	RNA-seq of B73: fully expanded third leaves 24 h	.fastq	NCBI Sequence Read Archive (http://identifiers.org/ncbi/insdc.sra:SRR11442837)
Data file 9	RNA-seq of B73: fully expanded third leaves 27 h	.fastq	NCBI Sequence Read Archive (http://identifiers.org/ncbi/insdc.sra:SRR11442870)
Data file 10	RNA-seq of B73: fully expanded third leaves 30 h	.fastq	NCBI Sequence Read Archive (http://identifiers.org/ncbi/insdc.sra:SRR11442869)
Data file 11	RNA-seq of B73: fully expanded third leaves 33 h	.fastq	NCBI Sequence Read Archive (http://identifiers.org/ncbi/insdc.sra:SRR11442906)
Data file 12	RNA-seq of B73: fully expanded third leaves 36 h	.fastq	NCBI Sequence Read Archive (http://identifiers.org/ncbi/insdc.sra:SRR11442905)
Data file 13	RNA-seq of B73: fully expanded third leaves 39 h	.fastq	NCBI Sequence Read Archive (http://identifiers.org/ncbi/insdc.sra:SRR11442904)
Data file 14	RNA-seq of B73: fully expanded third leaves 42 h	.fastq	NCBI Sequence Read Archive (http://identifiers.org/ncbi/insdc.sra:SRR11442903)
Data file 15	RNA-seq of B73: fully expanded third leaves 45 h	.fastq	NCBI Sequence Read Archive (http://identifiers.org/ncbi/insdc.sra:SRR11442902)
Data file 16	RNA-seq of B73: fully expanded third leaves 48 h	.fastq	NCBI Sequence Read Archive (http://identifiers.org/ncbi/insdc.sra:SRR11442901)
Data file 17	RNA-seq of B73: fully expanded third leaves 51 h	.fastq	NCBI Sequence Read Archive (http://identifiers.org/ncbi/insdc.sra:SRR11442900)
Data file 18	RNA-seq of B73: fully expanded third leaves 54 h	.fastq	NCBI Sequence Read Archive (http://identifiers.org/ncbi/insdc.sra:SRR11442899)
Data file 19	RNA-seq of B73: fully expanded third leaves 57 h	.fastq	NCBI Sequence Read Archive (http://identifiers.org/ncbi/insdc.sra:SRR11442898)
Data file 20	RNA-seq of B73: fully expanded third leaves 60 h	.fastq	NCBI Sequence Read Archive (http://identifiers.org/ncbi/insdc.sra:SRR11442897)
Data file 21	RNA-seq of B73: fully expanded third leaves 63 h	.fastq	NCBI Sequence Read Archive (http://identifiers.org/ncbi/insdc.sra:SRR11442895)
Data file 22	RNA-seq of B73: fully expanded third leaves 66 h	.fastq	NCBI Sequence Read Archive (http://identifiers.org/ncbi/insdc.sra:SRR11442894)
Data file 23	RNA-seq of B73: fully expanded third leaves 69 h	.fastq	NCBI Sequence Read Archive (http://identifiers.org/ncbi/insdc.sra:SRR11442893)
Data file 24	RNA-seq of B73: fully expanded third leaves 72 h	.fastq	NCBI Sequence Read Archive (http://identifiers.org/ncbi/insdc.sra:SRR11442892)
Data file 25	RNA-seq of Yugu1: fully expanded third leaves 3 h	.fastq	NCBI Sequence Read Archive (http://identifiers.org/ncbi/insdc.sra:SRR11442861)
Data file 26	RNA-seq of Yugu1: fully expanded third leaves 6 h	.fastq	NCBI Sequence Read Archive (http://identifiers.org/ncbi/insdc.sra:SRR11442860)
Data file 27	RNA-seq of Yugu1: fully expanded third leaves 9 h	.fastq	NCBI Sequence Read Archive (http://identifiers.org/ncbi/insdc.sra:SRR11442858)
Data file 28	RNA-seq of Yugu1: fully expanded third leaves 12 h	.fastq	NCBI Sequence Read Archive (http://identifiers.org/ncbi/insdc.sra:SRR11442857)
Data file 29	RNA-seq of Yugu1: fully expanded third leaves 15 h	.fastq	NCBI Sequence Read Archive (http://identifiers.org/ncbi/insdc.sra:SRR11442856)
Data file 30	RNA-seq of Yugu1: fully expanded third leaves 18 h	.fastq	NCBI Sequence Read Archive (http://identifiers.org/ncbi/insdc.sra:SRR11442855)
Data file 31	RNA-seq of Yugu1: fully expanded third leaves 21 h	.fastq	NCBI Sequence Read Archive (http://identifiers.org/ncbi/insdc.sra:SRR11442854)
Data file 32	RNA-seq of Yugu1: fully expanded third leaves 24 h	.fastq	NCBI Sequence Read Archive (http://identifiers.org/ncbi/insdc.sra:SRR11442853)
Data file 33	RNA-seq of Yugu1: fully expanded third leaves 27 h	.fastq	NCBI Sequence Read Archive (http://identifiers.org/ncbi/insdc.sra:SRR11442852)
Data file 34	RNA-seq of Yugu1: fully expanded third leaves 30 h	.fastq	NCBI Sequence Read Archive (http://identifiers.org/ncbi/insdc.sra:SRR11442851)
Data file 35	RNA-seq of Yugu1: fully expanded third leaves 33 h	.fastq	NCBI Sequence Read Archive (http://identifiers.org/ncbi/insdc.sra:SRR11442850)
Data file 36	RNA-seq of Yugu1: fully expanded third leaves 36 h	.fastq	NCBI Sequence Read Archive (http://identifiers.org/ncbi/insdc.sra:SRR11442849)
Data file 37	RNA-seq of Yugu1: fully expanded third leaves 39 h	.fastq	NCBI Sequence Read Archive (http://identifiers.org/ncbi/insdc.sra:SRR11442847)
Data file 38	RNA-seq of Yugu1: fully expanded third leaves 42 h	.fastq	NCBI Sequence Read Archive (http://identifiers.org/ncbi/insdc.sra:SRR11442846)
Data file 39	RNA-seq of Yugu1: fully expanded third leaves 45 h	.fastq	NCBI Sequence Read Archive (http://identifiers.org/ncbi/insdc.sra:SRR11442845)
Data file 40	RNA-seq of Yugu1: fully expanded third leaves 48 h	.fastq	NCBI Sequence Read Archive (http://identifiers.org/ncbi/insdc.sra:SRR11442844)
Data file 41	RNA-seq of Yugu1: fully expanded third leaves 51 h	.fastq	NCBI Sequence Read Archive (http://identifiers.org/ncbi/insdc.sra:SRR11442843)
Data file 42	RNA-seq of Yugu1: fully expanded third leaves 54 h	.fastq	NCBI Sequence Read Archive (http://identifiers.org/ncbi/insdc.sra:SRR11442842)
Data file 43	RNA-seq of Yugu1: fully expanded third leaves 57 h	.fastq	NCBI Sequence Read Archive (http://identifiers.org/ncbi/insdc.sra:SRR11442841)
Data file 44	RNA-seq of Yugu1: fully expanded third leaves 60 h	.fastq	NCBI Sequence Read Archive (http://identifiers.org/ncbi/insdc.sra:SRR11442840)
Data file 45	RNA-seq of Yugu1: fully expanded third leaves 63 h	.fastq	NCBI Sequence Read Archive (http://identifiers.org/ncbi/insdc.sra:SRR11442839)
Data file 46	RNA-seq of Yugu1: fully expanded third leaves 66 h	.fastq	NCBI Sequence Read Archive (http://identifiers.org/ncbi/insdc.sra:SRR11442838)
Data file 47	RNA-seq of Yugu1: fully expanded third leaves 69 h	.fastq	NCBI Sequence Read Archive (http://identifiers.org/ncbi/insdc.sra:SRR11442872)
Data file 48	RNA-seq of Yugu1: fully expanded third leaves 72 h	.fastq	NCBI Sequence Read Archive (http://identifiers.org/ncbi/insdc.sra:SRR11442871)
Data file 49	RNA-seq of BTx623: fully expanded third leaves 3 h	.fastq	NCBI Sequence Read Archive (http://identifiers.org/ncbi/insdc.sra:SRR11442891)
Data file 50	RNA-seq of BTx623: fully expanded third leaves 6 h	.fastq	NCBI Sequence Read Archive (http://identifiers.org/ncbi/insdc.sra:SRR11442890)
Data file 51	RNA-seq of BTx623: fully expanded third leaves 9 h	.fastq	NCBI Sequence Read Archive (http://identifiers.org/ncbi/insdc.sra:SRR11442889)
Data file 52	RNA-seq of BTx623: fully expanded third leaves 12 h	.fastq	NCBI Sequence Read Archive (http://identifiers.org/ncbi/insdc.sra:SRR11442888)
Data file 53	RNA-seq of BTx623: fully expanded third leaves 15 h	.fastq	NCBI Sequence Read Archive (http://identifiers.org/ncbi/insdc.sra:SRR11442887)
Data file 54	RNA-seq of BTx623: fully expanded third leaves 18 h	.fastq	NCBI Sequence Read Archive (http://identifiers.org/ncbi/insdc.sra:SRR11442886)
Data file 55	RNA-seq of BTx623: fully expanded third leaves 21 h	.fastq	NCBI Sequence Read Archive (http://identifiers.org/ncbi/insdc.sra:SRR11442884)
Data file 56	RNA-seq of BTx623: fully expanded third leaves 24 h	.fastq	NCBI Sequence Read Archive (http://identifiers.org/ncbi/insdc.sra:SRR11442883)
Data file 57	RNA-seq of BTx623: fully expanded third leaves 27 h	.fastq	NCBI Sequence Read Archive (http://identifiers.org/ncbi/insdc.sra:SRR11442882)
Data file 58	RNA-seq of BTx623: fully expanded third leaves 30 h	.fastq	NCBI Sequence Read Archive (http://identifiers.org/ncbi/insdc.sra:SRR11442881)
Data file 59	RNA-seq of BTx623: fully expanded third leaves 33 h	.fastq	NCBI Sequence Read Archive (http://identifiers.org/ncbi/insdc.sra:SRR11442880)
Data file 60	RNA-seq of BTx623: fully expanded third leaves 36 h	.fastq	NCBI Sequence Read Archive (http://identifiers.org/ncbi/insdc.sra:SRR11442879)
Data file 61	RNA-seq of BTx623: fully expanded third leaves 39 h	.fastq	NCBI Sequence Read Archive (http://identifiers.org/ncbi/insdc.sra:SRR11442878)
Data file 62	RNA-seq of BTx623: fully expanded third leaves 42 h	.fastq	NCBI Sequence Read Archive (http://identifiers.org/ncbi/insdc.sra:SRR11442877)
Data file 63	RNA-seq of BTx623: fully expanded third leaves 45 h	.fastq	NCBI Sequence Read Archive (http://identifiers.org/ncbi/insdc.sra:SRR11442876)
Data file 64	RNA-seq of BTx623: fully expanded third leaves 48 h	.fastq	NCBI Sequence Read Archive (http://identifiers.org/ncbi/insdc.sra:SRR11442875)
Data file 65	RNA-seq of BTx623: fully expanded third leaves 51 h	.fastq	NCBI Sequence Read Archive (http://identifiers.org/ncbi/insdc.sra:SRR11442873)
Data file 66	RNA-seq of BTx623: fully expanded third leaves 54 h	.fastq	NCBI Sequence Read Archive (http://identifiers.org/ncbi/insdc.sra:SRR11442868)
Data file 67	RNA-seq of BTx623: fully expanded third leaves 57 h	.fastq	NCBI Sequence Read Archive (http://identifiers.org/ncbi/insdc.sra:SRR11442868)
Data file 68	RNA-seq of BTx623: fully expanded third leaves 60 h	.fastq	NCBI Sequence Read Archive (http://identifiers.org/ncbi/insdc.sra:SRR11442866)
Data file 69	RNA-seq of BTx623: fully expanded third leaves 63 h	.fastq	NCBI Sequence Read Archive (http://identifiers.org/ncbi/insdc.sra:SRR11442865)
Data file 70	RNA-seq of BTx623: fully expanded third leaves 66 h	.fastq	NCBI Sequence Read Archive (http://identifiers.org/ncbi/insdc.sra:SRR11442864)
Data file 71	RNA-seq of BTx623: fully expanded third leaves 69 h	.fastq	NCBI Sequence Read Archive (http://identifiers.org/ncbi/insdc.sra:SRR11442863)
Data file 72	RNA-seq of BTx623: fully expanded third leaves 72 h	.fastq	NCBI Sequence Read Archive (http://identifiers.org/ncbi/insdc.sra:SRR11442862)

## Limitations

- This is RNA-seq analysis of leaf blade tissue from young plants (15–20 days after planting). Care should be taken in directly extrapolating the transcriptome profiles to other tissues and/or older plants.
- The plants were grown in a growth chamber under cool white fluorescent lighting and under well-controlled temperatures. These conditions should be taken into account when making comparisons to plants grown in the field under natural sunlight and temperature.
- The goal of the RNA-seq design was to capture transcript levels from expressed genes, not to describe the full range of transcript splice variants.

## Data Availability

All the data described in this Data note can be freely and openly accessed at the NCBI Sequence Read Archive. All the runs are listed with resolving links in Table 1, and all are gathered together under NCBI Sequence Read Archive study accession SRP254420 [3]. Please see Table 1 and reference Lai et al. [1] for details and links to the data.

## Abbreviations

NCBI: National Center for Biotechnology Information
PE50: Paired-end sequenced with 50 cycles
